# Flood risk reduction in the northern coast of Central Java Province, Indonesia: An application of stakeholder’s analysis

**DOI:** 10.4102/jamba.v11i1.660

**Published:** 2019-07-18

**Authors:** Muzakar Isa, Akhmad Fauzi, Indah Susilowati

**Affiliations:** 1 Faculty of Economics, Universitas Muhammadiyah Surakarta, Jawa Tengah, Indonesia; 2 Department of Resource and Environmental Economics, Bogor Agricultural University, Bogor, Indonesia; 3 Faculty of Economics and Business, Diponegoro University, Jawa Tengah, Indonesia

**Keywords:** coastal, flood, risk, stakeholders, interest, convergent

## Abstract

The northern coast of Central Java Province, Indonesia, is considered as the critical area of flooding. The area always suffers from flooding because of heavy rainfall and/or sea level rise. Flooding brings a lot of consequences, including people’s suffering, property damage and property loss. A number of efforts have been carried out to manage flood problems, yet the achievement is incomplete without stakeholder involvement. Stakeholders have a very important role in flood risk reduction. A common understanding on the existence and role of stakeholders is important in achieving community resilience. The aim of this article was to analyse interest and cooperation among stakeholders in flood risk reduction. In-depth interviews were conducted to identify and analyse interest and influence of stakeholders in relation to flood risk reduction. MIC-MAC (Cross-impact Matrix Multiplication Applied to Classification) and MACTOR (Matrix of Alliance and Conflict: Tactics, Objectives, and Recommendations) methods were employed to analyse collected data. The results of this study indicated that restoration and humanity are the key objectives with major influence in lowering flood risk. The objectives are critical for the success of flood risk reduction efforts. The Disaster Management Agency is identified as the main stakeholder with the most important role in reducing flood risk in Central Java Province.

## Introduction

The Indonesia Disaster Data and Information shows a long-term upward trend in the number of floods in Indonesia, in which 5233 occurrences or approximately 38.99% of the total number of natural disasters occurred between 1815 and 2015. From 2011 to 2015, 368 cases of flooding in the Central Java Province, Indonesia, were reported, and the number has been continuously rising ever since (Isa, Sugiyanto & Susilowati 2018). In addition, flood hazards brought huge impacts, 58 people died, 191 422 residents were evacuated, 31.012 hectares was destroyed, 139 km of roads were broken and 1104 houses were heavily damaged (Isa et al. 2018). The figure could be significantly higher if the property damaged by surface water flooding was also taken into account.

Flood risks have a propensity to extend massively in the future, especially because of the impacts of climate change (Pitt 2008). As flooding is a hazard that could possibly occur because of weather conditions (heavy rainfall) coupled with other causes (e.g. inadequate drainage and overflowing river banks), a higher intensity and frequency of such weather extremes are likely to increase the risk of flooding. Fowler and Wilby (2010) noted a rising trend in rainfall intensity because of an increase in heavy rainfall. In fact, the recent prevalence of floods in the Central Java Province is emanating from heavy rainfall and is likely to exacerbate in future (Isa 2016; Isa et al. 2018).

Flooding can have a critical impact on communities, both directly and indirectly (Isa et al. 2013). Infrastructural damage, ineffective workdays, shortened business hours and community inconvenience are several short-term impacts of flooding in the coastal zones. Long-term impacts include disrupted cash flow and income loss. Although direct impacts are often highlighted, indirect impacts of flooding could also bring major effects on communities. Woodman (2008) identified that 53% off-days for employees, 38% of premises flooded (offices, shops, etc.) and 27% of disrupted supplies became the main impacts of flooding endured by 255 business actors as the respondents of a study in 2007. These findings suggest that the indirect impacts of flooding are actually higher than the direct impacts.

In order to reduce the impacts, Morton et al. (2011) asserted that it will be optimal if flood risk reduction is done collectively rather than separately. Therefore, stakeholders’ participation in flood risk reduction becomes an important aspect in building resilient communities. The need for building resilient communities, which can bounce back from the impacts of such hazards, has become a focal point of discussion during the recent years (Cutter et al. 2008; Manyena 2006; Paton 2006).

Reed et al. (2009) explained that the term ‘stakeholder’ emerged in the 17th century. Stakeholder is defined as a group or individual within an organisation (Gaur 2013). In reducing flood risk, this term is used to describe individuals and communities or organisations that are affected by activities or policies, in which a party does not always receive a fair impact (Raman, Ojo & Dorasamy 2015). Some parties may bear the costs, and some will gain benefits from an activity or policy (Race & Millar 2008). Bryson (2003) defines stakeholders as any individual or group that can impact or be impacted by the success of an organisation’s goals. It may be based on a policy, programme or development activity. Stakeholders can be individuals, communities, socio-economic groups or institutions at every dimension and level of society (Lienert & Schnetzer 2013). Each of these groups has resources and needs to be represented in the decision-making process of development activities. The decision-making process cannot be effectively implemented by only one particular group (Gonsalves et al. 2005).

Crosby (1991) explained that there are two important things related to the existence of stakeholders in the implementation of flood risk reduction, namely the classification and the participation of stakeholders. Based on the classification, stakeholders are classified as main stakeholders, supporting stakeholders and stakeholders with either formal or informal interests. The main stakeholders are those who receive positive and negative impacts from an activity. The supporting stakeholders are those who become intermediaries in assisting the process of delivering activities. The supporting stakeholders can be classified as funders, executors, supervisors, government agencies, non-governmental organisations (NGOs) and private sectors. And the last group of stakeholders is individuals or key groups with either formal or informal interests. These stakeholders have strong influence or importance in relation to the problem, need and attention to the smooth running of activities. Meanwhile, the participation of stakeholders is a medium to achieve the objectives in the implementation of activities. Through this participation, it is expected that an action plan can be formulated and simultaneously implemented at once (Iqbal 2007).

According to Iqbal (2007), there are four reasons to highlight the significance of participation in supporting the success of a programme and/or activity of flood risk reduction. Firstly, participation is needed to improve programme development plans and/or activities in general and to prioritise activities, in particular of flood risk reduction. Secondly, participation has a purpose for the implementation of activities in accordance with the stakeholder objectives. Thirdly, participation is needed to ensure the sustainability of flood risk reduction activities. Fourthly, participation can improve equality in the implementation of flood risk reduction activities.

Freeman advances that stakeholders are an important group for the continuity and success of a corporation and/or community in achieving their goals (Miles 2012; Reed et al. 2009). Generally, the stages of an activity, ranging from planning to evaluation, are carried out only based on custom and the existing or established job description. As a result, it is frequently less appropriate with requirements in the field. Stakeholder analysis becomes one of the necessary alternatives to encourage the participation of each stakeholder (Putterman 2013).

The aim of this study was to identify stakeholders’ interests or key variables in conducting flood risk reduction activities and to analyse stakeholder relationships in conducting flood risk reduction.

## Materials and methods

### Study area

**FIGURE 1:**
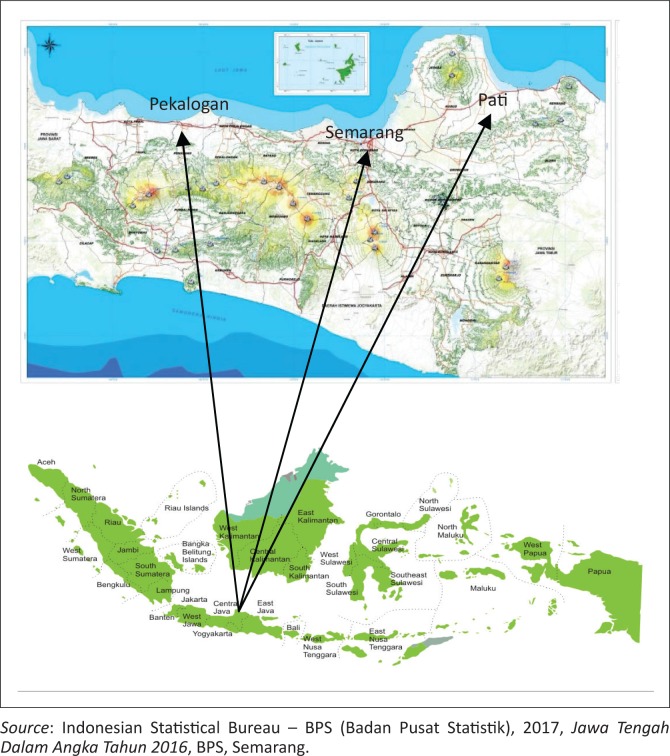
Location of the study sites.

This study was conducted in three sites that represent the eastern, central and western part of the northern coast of Central Java Province; they are Pati Regency, Pekalongan Regency and Semarang City (Figure 1). The Central Java Province is one of the 34 provinces of Indonesia. It has an area of 3 254 412 hectares, constituting 1.70% of the total area of Indonesia (BPS-Statistics Indonesia 2017).

**FIGURE 2:**
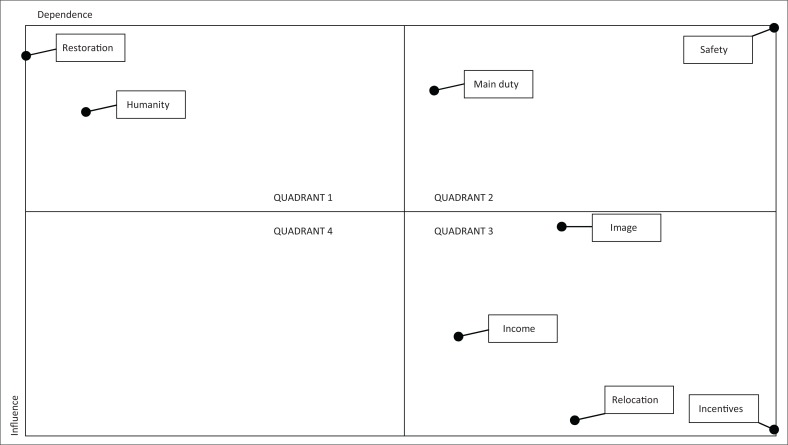
Map influence and interdependence between the objectives of stakeholders.

Central Java is bordered by the Indian Ocean and Yogyakarta to the south, West Java to the west, East Java to the east and the Java Sea to the north. Central Java is located between 5^°^ 40’ and 8^°^ 30’ South Latitude and between 108^°^ 30’ and 111^°^ 30’ East Longitude. More than 53% of the Central Java region is lowland. The lowlands lie on the north coast and the west coast. The north coast is more vulnerable to flooding. The flooding is caused by high rainfall, overflow of the rivers and the damage of dams and/or water gates. High intensity flooding occurs in the Pati Regency, Pekalongan Regency and the Semarang City (Isa et al. 2018). Several major rivers pass through these areas making them more vulnerable to flooding. The land-use conversion into residential areas, agricultural expansion and industrial development in the lowlands contribute to the degradation of coastal areas in the Pati Regency, Pekalongan Regency and Semarang City. Figure 2 shows the location of the study sites.

### Data processing and analyses

Primary data were collected using questionnaires. Structural analysis was performed with the analytical tools such as MIC-MAC (Cross-impact Matrix Multiplication Applied to Classification) and MACTOR (Matrix of Alliance and Conflict: Tactics, Objectives, and Recommendations).

Cross-impact Matrix Multiplication Applied to Classification is an analytical tool used to analyse the drive power and dependence power of factors and various objectives of stakeholders. The Cross-impact Matrix Multiplication Applied to Classification principle is based on the multiplication properties of matrices. Analysis using MIC-MAC is performed to identify the key factors that drive the system in various categories. Based on their drive power and dependence power, the factors have been classified into four categories which are autonomous factors, linkage factors, dependent factors and independent factors (Rajesh et al. 2013). The results of the analysis show the various objectives of stakeholders as the main objectives in reducing flood risk (Mandal & Deshmukh 1994).

Matrix of Alliance and Conflict: Tactics, Objectives, and Recommendations is an analytical tool used to analyse the influences and interests (objectives) of stakeholders (Mangifera & Isa, 2019). This method is very broad because it can be used for up to 20 related purposes and yet is simple and accessible (Godet 1991; Rees & MacDonell 2017). Matrix of Alliance and Conflict: Tactics, Objectives, and Recommendations includes several stages of matrix preparation, namely: (1) determination of key variables and relevant actors, (2) preparation of a table of actors, (3) preparation of strategic issues and objectives, (4) determination of actors and strategic objectives in the matrix, (5) calculation of convergence and divergence matrices as performed in three stages, (6) calculation of the direct and indirect relationship of the power matrix, (7) calculation of the position matrix value and (8) analysis of strategic recommendations of each actor (Godet 1991).

### Ethical considerations

This article followed all ethical standards for research without direct contact with human or animal subjects.

## Results and discussion

In their flood risk reduction efforts, stakeholders have various strategic roles. Their roles are explained based on the analysis of objectives and relationships among them. In this research, 22 individuals represented the main actors in flood risk reduction.

The stakeholder analysis consisted of three stages that start initially with the identification of objectives of a key person in flood risk reduction. It is the most important stage for the whole process. The second stage is the explanation of relationships among stakeholders’ objectives. It explains the relationship between the goals of each stakeholder in reducing the risk of flooding, and the last stage is the identification of key stakeholders’ objectives in flood risk reduction.

### Stakeholders’ objectives

The list of stakeholders’ objectives was further processed based on structural analysis with MIC-MAC as the analytical tool. This analysis was used to describe the relationship among the objectives of each stakeholder in conducting the related activities (Wajdi, Isa, & Setyawan 2015).

The results of MIC-MAC analysis on the stakeholders’ objectives in flood risk reduction were classified into four quadrants (Isa, Sugiyanto & Susilowati 2015):

- Quadrant 1 is an autonomous factor (weak influence driver – weak dependent). This quadrant includes objectives that have a weak influence and dependence. The objective is less related to flood risk reduction and may have few relationships that will be eliminated from the objectives of stakeholders in flood risk reduction.
- Quadrant 2 is dependent factors (weak influence – strongly dependent). It includes objectives that have weak influence and strong dependence. The objectives in this quadrant are non-independent ones.
- Quadrant 3 is a linkage factor (strong influence – strongly dependent). It includes objectives that have a strong influence and strong dependence. They should be determined meticulously as the relationship between goals is unstable.
- Quadrant 4 is an independent factor (strong influence – weak dependent). This quadrant includes objectives that have strong strength and weak dependence. The objectives are the key factors in flood risk reduction.

The objectives in the upper-left quadrant (restoration and humanity) imply the high influence and low dependence of other variables in flood risk reduction. These objectives have major influence in reducing flood risk. They, thus, become the key objectives that are critical in the success of flood risk reduction efforts. Restoration enables stakeholders to undertake the development, maintenance and improvement of natural resources and/or infrastructures, such as rivers, embankments and floodgates, which have been proven to lower the risk of flooding, while humanity raises awareness and empathy to others. These two objectives have the greatest impact in reducing flood risk.

The second most important quadrant is the upper-right quadrant. The two objectives included in this quadrant are main duty and safety. The objective of carrying out the main duty is important for government agencies because it is their main activity as well as a major indicator of the performance of their agencies. The objective of promoting people’s safety is also an important goal for an individual or group or organisation in reducing flood risk.

The lower-right quadrant is dependent on weak driver and strongly dependent variables. The objective of this quadrant is the element of awareness, which is a non-independent factor. Image, income, relocation and incentives are also classified in this quadrant. They are the objectives of several stakeholders in conducting flood risk reduction activities.

**FIGURE 3:**
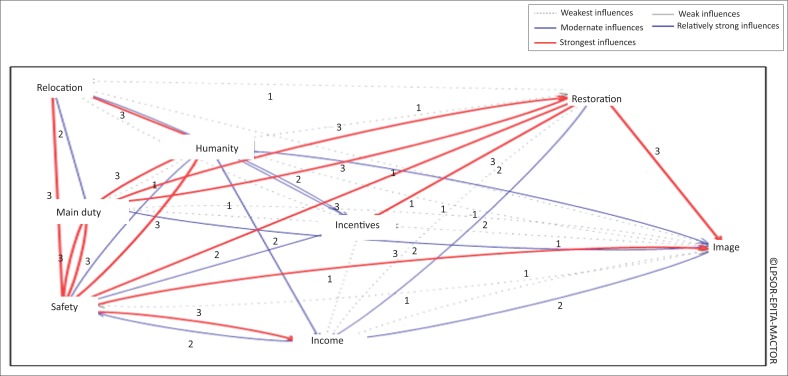
Relationship between stakeholders’ objectives.

Figure 2 shows the objectives that strongly influence other objectives such as restoration and humanity. The objectives of stakeholders to restore and safeguard the public and environment have the ability to influence other objectives such as the implementation of the main duty, image, safety, income, incentives and relocation. This is confirmed by the research of Mandal and Deshmukh (1994) and Isa et al. (2015) which show that stakeholders have different linked objectives.

### Relationship between stakeholders

Stakeholders involved in the activities of flood risk reduction have different influences and interests (objectives). Based on the identification and analysis of stakeholders, flood risk reduction activities in the northern coastal area of Central Java Province, Indonesia, were conducted by the Provincial Disaster Management Agency (Badan Penanggulangan Bencana Daerah [BPBD] Jateng), Semarang City Disaster Management Agency (Badan Penanggulangan Bencana Daerah [BPBD] Semarang), Pati Regional Disaster Management Agency (Badan Penanggulangan Bencana Daerah [BPBD] Pati), Pekalongan Regional Disaster Management Agency (BPBD Pekalongan), Regional Development Planning Agency (Badan Perencanaan Pembangunan Daerah[APPEDA]), Public Health Office (Dinas Kesehatan), Public Works Service (Dinas Pekerjaan Umum), Environmental Services (Dinas Lingkungan Hidup), businessmen, village heads, Search and Rescue (SAR) Team (Tim SAR), Indonesian Military and Indonesian Police (Indonesian National Armed Forces-Indonesian National Police [TNI and POLRI]), Indonesian Red Cross, farmers, volunteers, fisherman, village community leaders, local community (Masyarakat), Non-government Organisation (NGO) (community), Universities (university), business actors and journalists.

Stakeholders in flood risk reduction can be categorised into three groups (Reed et al. 2009), namely: (1) beneficiaries – comprised of individuals, organisations or community groups exposed to flood risk, either directly or indirectly, including farmers, fishermen, the community and businessmen; (2) intermediaries – comprised of organisations, community groups or individuals who can provide strategy and/or facility in the reduction of flood risk, including SAR Team, TNI and POLRI, Indonesian Red Cross, NGO (community) and universities that have programmes related to flood risk reduction; (3) policy-makers – comprised of institutions, such as the Provincial Disaster Management Agency, City Disaster Management Agency, Regional Disaster Management Agency, Regional Development Planning Agency (BAPPEDA), Public Health Office, Public Works Service (DPU), and Environmental Services (DinLH), authorised to make decisions on a legal basis.

**FIGURE 4:**
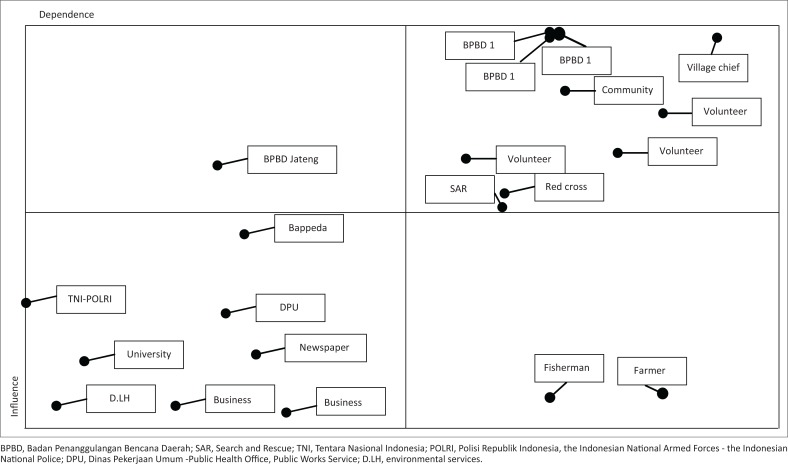
Map of influence and interdependence between stakeholders.

The effects and interests of stakeholders in flood risk reduction are illustrated in Figure 4.

Based on Figure 4, the Provincial Disaster Management Agency (BPBD Jateng) is the only stakeholder in quadrant 1. It means BPBD of the Central Java Province is the stakeholder that has the highest competitiveness, a high influence and low dependence. Stakeholders in this quadrant are influential stakeholders but have a low level of importance in policy goals and outcomes. Stakeholders in quadrant 2 are the Semarang City Disaster Management Agency (BPBD Semarang), Pati Regional Disaster Management Agency (BPBD Pati), Pekalongan Regional Disaster Management Agency (BPBD Pekalongan), Regional Development Planning Agency (BAPPEDA), Public Health Office (Dinas Kesehatan), environmental services (DinLH), village heads, SAR Team, Indonesian Red Cross, volunteers and NGOs involved in disaster risk management. These stakeholders are those with a high influence and high dependence. Stakeholders in quadrant 3 are those having low influence and high dependence to succeed in reducing flood risk. These are farmers and fishermen with a high dependence in flood risk reduction activities but who have low influence. Lastly, stakeholders in quadrant 4 are the Regional Development Planning Agency (BAPPEDA), Public Works Service (DPU), TNI and POLRI, universities, businessmen and journalists. These stakeholders are those with low influence and low dependence. They are stakeholders who are naturally and automatically associated with the reduction of flood risk.

**FIGURE 5:**
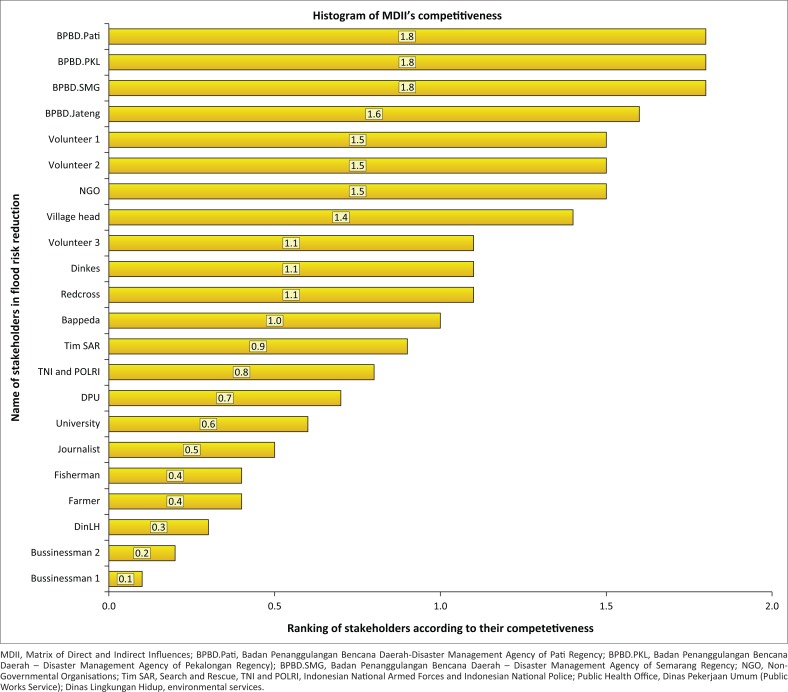
Diagram of competitiveness of stakeholders in flood risk reduction.

The influence and significance of stakeholders show their competitiveness in reducing the risk of flooding in the study sites. The competitiveness is demonstrated in Figure 5.

The Disaster Management Agency of Semarang City (BPBD Semarang), Disaster Management Agency of Pati Regency (BPBD Pati) and Disaster Management Agency of Pekalongan Regency (BPBD Pekalongan) are the institutions responsible for disaster risk reduction at regional level and have the highest impact. It means that these institutions have an important role in reducing flood risk in Central Java Province. The BPBD Central Java Province ranks second, followed by community leaders as stakeholders who have a high competitiveness in reducing flood risk in the Central Java Province.

**FIGURE 6:**
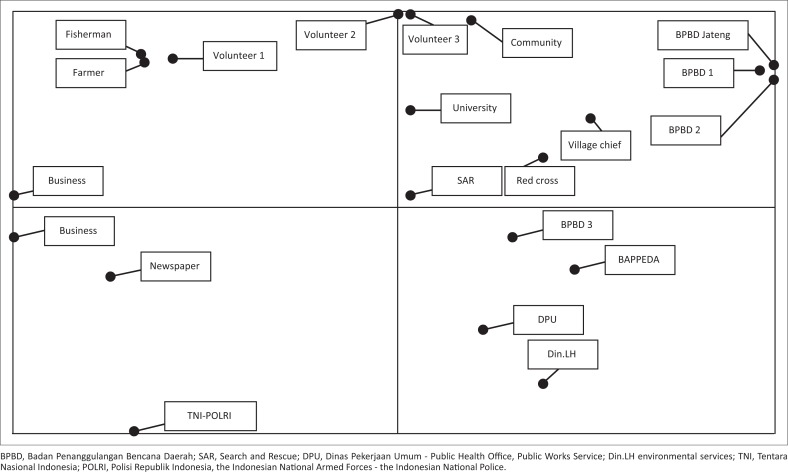
Convergent actor map in flood risk reduction.

The other major findings of stakeholder analysis are convergence and divergence (Ackermann & Eden 2011). The convergence matrix is divided into three orders, namely the convergence of objectives between the actors which is used to identify the general position of an actor, either pro or contra. The closer the actors is the stronger the convergence (Figure 6). The neutral position is not included. The second order is a convergence matrix associated with the actor X objective that is calculating the average convergence intensity between two actors which have the same level, either pro or contra. The matrix calculation focuses on the intensity of the alliance with the preference of the objectives of some actors. The third order is the matrix calculation using the weighted value, which is the weighted position of the actor X objectives. This order identifies the number of alliances and also considers the actor’s preference in objectives and competitiveness. Values represent the level of convergence in which the higher the intensity, the more the actors will have a common interest.

**FIGURE 7:**
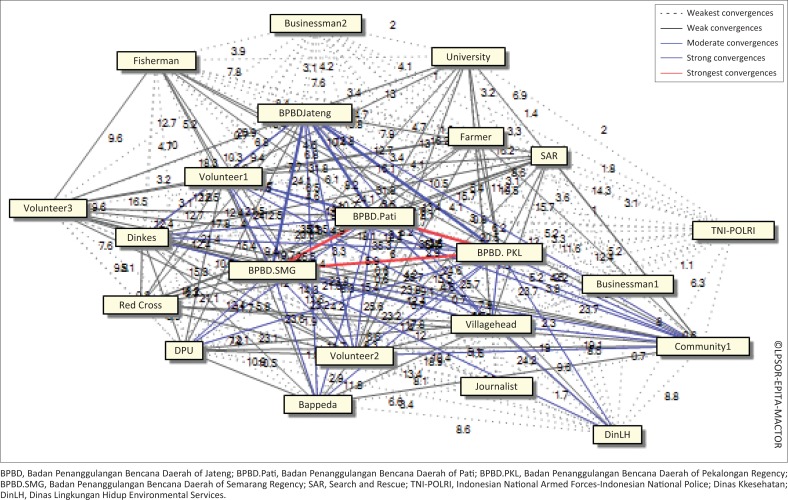
Actor relationships in reducing flood risk.

Very strong convergence occurs among the Provincial Disaster Management Agency (BPBD Jateng), Semarang City Disaster Management Agency (BPBD Semarang), Pati Regional Disaster Management Agency (BPBD Pati), Pekalongan Regional Disaster Management Agency (BPBD Pekalongan), Regional Development Planning Agency (BAPPEDA), Public Health Office, Public Works Service (DPU), environmental services, businessmen, village heads, SAR Team, TNI and POLRI, Indonesian Red Cross (PMI), village community leaders, Community, NGO, universities and business actors (Figure 7). Stakeholders are important actors in flood risk reduction in the northern coastal area of the Central Java Province because they have the same interest or purpose and a relatively close relationship. Meanwhile, businessmen, SAR, PMI and TNI-POLRI are classified as stakeholders with a low convergence. They are not key elements in reducing the risk of flooding in this region. The level of convergence is related to the magnitude of stakeholder influence and importance in the activities of flood risk reduction in the northern coastal region.

Based on the actor’s preference in objectives and competitiveness, the actors involved in reducing flood risk groups are grouped into four major groups. The first group consists of BPBD Central Java Province, BPBDs at regional level, Health Office, Public Works Service (DPU), environmental services, village heads, SAR and Indonesian Red Cross. The second group consists of universities, farmers, communities, fishermen, community leaders and NGOs (community). The third group includes the business actors and journalists, and the fourth group is the TNI-POLRI.

The next finding is divergence. The results of MACTOR analysis show that stakeholders with a high potential of divergence are BPBD, farmers, fishermen and businessmen. The result indicates that these stakeholders have a relatively high probability of conflict or goal difference. On the contrary, university, TNI-POLRI, PMI and SAR have a low probability of conflict. In fact, their position is neutral because they have indirect interests in flood risk reduction activities.

## Conclusion

Not all actors involved in flood management have an important role in reducing flood risk. Each stakeholder has different objectives in conducting flood risk reduction activities; they are restoration, humanity, safety, income, image, main duty, incentive and relocation. Restoration and humanity are two objectives that have a major influence in lowering flood risk. These key objectives are critical in supporting the success of flood risk reduction. Restoration encourages stakeholders to undertake the development, maintenance and improvement of natural resources and/or infrastructure, such as rivers, embankments and floodgates, which may lower the risk of flooding. Meanwhile, humanity raises the awareness and empathy to others. In general, stakeholders in flood risk reduction can be grouped into three, namely beneficiaries, intermediaries and policy-makers. The Disaster Management Agency (BPBD) as the institution of policy-makers for disaster risk reduction at the regional level appears to have the highest role. The Disaster Management Agency (BPBD) has an important role in reducing flood risk in Central Java Province.
